# Association between vitamin D receptor gene polymorphisms, inflammatory cytokines (IL-1, IL-6, and IL-10), and fracture healing in sports-related injuries

**DOI:** 10.5937/jomb0-56860

**Published:** 2025-09-05

**Authors:** Dan Wang

**Affiliations:** 1 Xijing University, Sports Education Center, Xian, Shaanxi, China

**Keywords:** bone mineral density, interleukins, IL-1, IL-6, IL-10, rehabilitation status, SRF, vitamin D receptors, vitamin D, mineralna gustina kostiju, interleukini, IL-1, IL-6, IL-10, status rehabilitacije, SRF, receptori za vitamin D, vitamin D

## Abstract

**Background:**

In this study, we analysed the correlation between polymorphisms in the Vitamin D receptors (VDR) gene at the Apa I and FoK I loci and sports-related fractures (SRF). We also examined the inflammatory cytokines IL-1, IL-6, and IL-10 to explore their potential role in fracture healing.

**Methods:**

A retrospective analysis was conducted on 110 patients with SRF admitted to our hospital from February 2023 to October 2023. Among these, 41 patients with poor fracture healing (research group) and 69 patients with good fracture healing (control group) were included. Serum levels of Vitamin D (VD), bone mineral density (BMD), and inflammatory cytokines (IL-1, IL-6, and IL-10) were measured to assess their relationship with fracture healing. Additionally, polymorphisms in the VDR gene at the Apa I and FoK I loci were analysed to determine the differences between the two groups.

## Abstract (sr)

**Uvod:**

U ovoj studiji je analizirana korelacija između polimorfizama gena receptora za vitamin D (VDR) na lokusima Apa I i FoK I i sportskih preloma (SRF). Takođe su ispitani inflamatorni citokini IL-1, IL-6 i IL-10 kako bi se istražila njihova potencijalna uloga u zarastanju preloma.

**Metode:**

Retrospektivna analiza je obuhvatila 110 pacijenata sa SRF koji su primljeni u našu bolnicu od februara 2023. do oktobra 2023. godine. Od toga, su u studiju uključeni 41 pacijent sa slabim zarastanjem preloma (grupa ispitanika) i 69 pacijenata sa dobrim zarastanjem preloma (kontrolna grupa). Mereni su serumski nivoi vitamina D (VD), mineralna gustina kostiju (BMD) i inflamatorni citokini (IL-1, IL-6 i IL-10) kako bi se procenila njihova povezanost sa zarastanjem preloma. Pored toga, analizirani su polimorfizmi gena VDR na lokusima Apa I i FoK I radi utvrđivanja razlika između ove dve grupe.

**Rezultati:**

Nivoi VD u grupi ispitanika su značajno niži nego u kontrolnoj grupi (P<0,05). Nivoi VD su bili u pozitivnoj korelaciji sa BMD u obe grupe (P<0,05). Analiza inflamatornih citokina pokazala je da su nivoi IL-1 i IL-6 značajno viši, dok su nivoi IL-10 niži u grupi ispitanika u poređenju sa kontrolnom grupom (P<0,05). U pogledu polimorfizma VDR, grupa ispitanika je imala veću učestalost FoK I CC genotipa i nižu učestalost TT genotipa (P<0,05), a nisu pronađene značajne razlike za Apa I polimorfizam (P>0,05).

**Zaključak:**

Polimorfizmi gena VDR, posebno na FoK I lokusu, usko su povezani sa napretkom rehabilitacije kod SRF. Pored toga, inflamatorna neravnoteža, koju karakterišu povišeni nivoi IL-1 i IL-6 i smanjeni nivoi IL-10, može doprineti slabom zarastanju preloma.

## Introduction

Sports-related fractures (SRF) are among the most common injuries seen in athletes and individuals who engage in physical activity. These fractures typically occur due to trauma, direct impact, or excessive muscle contractions during exercise [1]. While SRF can affect individuals of all ages, the risk tends to increase with age [2]. The incidence of fractures is parti cularly high in competitive sports, where the intensity of physical activity elevates the likelihood of injury [3]. Epidemiological data reveals that professional athletes account for approximately 50% of all SRF, while older people contribute to around 30% of this population [4]. Although bones are regenerative, the recovery process following a fracture generally extends beyond three months. Common sites of SRF include the femur, ankle joint, lumbar spine, and other regions, often leading to a significant loss of mobility and considerable disruption to daily life [5]. Conse qu ently, enhancing the rehabilitation process for these fra ctures has become a critical area of clinical research.

Vitamin D (VD) is a crucial trace element that plays a fundamental role in regulating calcium-phosphorus metabolism and maintaining bone health. A study by Wen Y et al. [6] confirmed that VD status is associated with skeletal muscle loss after ACL reconstruction, whereas Yoon S et al. [7] stated that VD is a potential nutritional factor that can significantly affect physical function and musculoskeletal injury in athletes. The Vitamin D receptor (VDR), a member of the steroid hormone/thyroid hormone receptor superfamily, functions as a nuclear transcription factor that regulates the differentiation and proliferation of various cell types [8]. VDR help increase the production of osteocalcin, promoting the secretion of cytokines by bone cells, which are essential for bone tissue formation and mineralisation [9]. Additionally, VDR mediates the active form of VD, 1,25-dihydroxy vitamin D3, to maintain calcium homeostasis and regulate immune function [10]. We conjecture that VDR gene polymorphisms may determine the state of skeletal rehabilitation in patients with SRF, but there is a lack of studies to confirm our view.

Emerging evidence suggests that cytokines such as Interleukin (IL)-1, IL-6, and IL-10 play a critical role in the inflammatory and healing processes associated with bone fractures. IL-1 and IL-6 are pro-inflammatory cytokines that are involved in bone resorption and the inflammatory response following injury, while IL-10 is an anti-inflammatory cytokine that helps modulate the immune response and promote tissue repair [11]. Understanding the interactions between VD, VDR, and these cytokines may provide valuable insights into improving fracture healing and rehabilitation outcomes.

This study aims to explore the relationship between VDR gene polymorphisms, inflammatory cytokines (IL-1, IL-6, IL-10), and the rehabilitation progress of SRF. These results will provide new objective references for future rehabilitation of SRF, thus optimising the patient’s prognosis.

## Materials and methods

### Subjects

First, we estimated the sample size of the study using the G-Power software: effect size=0.5, alpha err prob=0.05, power=0.9, and the estimation was that at least 34 study subjects were needed in each group. A retrospective analysis was conducted on 110 patients with SRF admitted to our hospital from February 2023 to October 2023. Among them, 41 patients with poor fracture healing were regarded as the research group, and 69 patients with good fracture healing were regarded as the control group. This study, approved by the Ethics Committee of the Sports Education Center, Xijing University, was conducted strictly following the *Helsinki Declaration*. Because the study was a retrospective analysis, the requirement for informed consent was waived.

### Eligibility and exclusion criteria

Inclusion criteria: Patients included were confirmed with SRF by imaging examinations (X-ray, CT or MRI to clarify the fracture, ask the patient about the aetiology of the fracture due to sports injury) (2), with age>18 years old and complete medical records. Exclusion criteria: Patients with autoimmune deficiency, administration of sex hormones, VD, glucocorticoids, or anti-osteoporosis drugs in recent 6 months, primary or metastatic vertebral tumours, organic diseases of vital organs such as liver and kidneys, or abnormal mental behaviours, were excluded. In addition, patients with comorbid acute or chronic inflammatory conditions were excluded.

### Criteria for poor healing

Poor healing includes delayed union and nonunion [12]. Delayed union refers to the fact that the fracture has not healed at the return visit 4 months after discharge, with mild decalcification of the fracture end, less callus, and obvious fracture line. Nonunion is defined as the separation of the fracture end, separation of the broken ends of the fractured bone, cherry blossom at the bone end, and atrophy and smoothness of both fractured ends.

### Sample collection

Fasting venous blood was collected from patients within 30 minutes of admission, collected in procoagulant tubes, and divided into three portions after standing for 30 minutes at room temperature.

### VD detection

VD levels were detected using liquid chromatography-tandem mass spectrometry (LC-MS/MS). Take 100–200 μL of serum, add methanol containing an isotopic internal standard (d3-25(OH)D_3_), and vortex to mix. Centrifuge (10,000 × g, 10 min) to remove precipitated proteins and take the supernatant. Using a VD solid-phase extraction column, interfering substances such as phospholipids were removed. The eluate was dried and re-solubilised with a mobile phase for online analysis. LC: reversed-phase column (C18 column, 2.1×50 mm, 1.7–2.6 mm particle size), 40–50 °C. Phase A: water (containing 0.1% formic acid or 5 mmol/L ammonium formate). Phase B: organic phase (methanol, comprising 0.1% formic acid). Gradient elution, 0.2–0.4 mL/min, injection volume 5–20 μL. MS/MS: Ion source was atmospheric pressure chemical ionisation (APCI), positive ion MS/MS: Ion source was atmospheric pressure chemical ionisation (APCI), positive ion mode. MS parameters: 25(OH)D_3_: m/z 401.3 → product ion m/z 383.3 (dehydration), 365.3 (secondary dehydration). 50–150 ms/channel. Stable isotope internal standards (e.g., d3-25(OH)D_3_) were added to correct for matrix effects and recovery differences. Intra/interday reproducibility (CV<15%) with recoveries of 85–115%.

### VDR polymorphism detection

Serum DNA was extracted and amplified using phenol-chloroform extraction. Shanghai Dynegene Technologies Co., Ltd., commissioned the primer sequence for design and construction (upstream sequence: 5’-CAACCAAGACAAGTACCGTACCGCGTCAGTGA-3’; downstream sequence: 5’-TGGCGGCAGCGGATGTACGTCTGC-3’), and the specific amplification product fragment was 1850 bp. VDR genotypes were analysed using polymerase chain reaction (PCR)-restriction fragment length polymorphism (RFLP). Reaction system: Taq DNA polymerase: 1 U (0.5 μL, 2 U/μL), dNTPs: 0.2 mmol/L (1 μL, 5 mmol/L), upstream and downstream primers: 0.5 μmol/L each (1 μL of each, 10 μmol/L), 10× PCR buffer: 2.5 μL (containing Mg^2+^ 15 mmol/L), template DNA: 1 μL (50–100 ng/μL), ddH_2_O: make up to 25 μL. The reaction conditions were 94°C for 5 minutes, 94°C for 30s, 61°C for 40s, and 72°C for 90s, for 35 cycles, followed by extension at 72°C for 10 minutes. Reaction system: Taq DNA polymerase: 1 U (0.5 μL, 2 U/μL), dNTPs: 0.2 mmol/L (1 μL, 5 mmol/L), upstream and downstream primers: 0.5 μmol/L each (1 μL of each, 10 μmol/L), 10× PCR buffer: 2.5 μL (containing Mg^2+^ 15 mmol/L), template DNA: 1 μL (50–100 ng/μL), ddH_2_O: make up to 25 μL. Validation of agarose gel electrophoresis: Gel preparation: 1.5% agarose (1.5 g agarose + 100 mL 1×TAE buffer), add 0.5 μg/mL ethidium bromide. Sampling volume: take 5 μL of PCR product and mix it with 1 μL of 6×sampling buffer. Electro phoresis conditions: 100 V, 20 min, 1× TAE buffer. Results observation: 1850 bp specific bands were detected by a gel imaging system. Enzyme digestion system: PCR product: 10 μL (about 200–500 ng DNA), BsmI endonuclease: 10 U (1 μL, 10 U/μL), 10 × buffer: 2 μL ddH_2_O: replenish to 20 μL, reaction conditions: incubation at 55°C for 4 h. Termination: inactivate the enzyme by heating at 80°C for 10 min.

After the reaction was terminated, the product was analysed using 1.5% agarose gel electrophoresis followed by ethidium bromide staining. The results were visualised with a gel imaging system based on DNA fragment length.

In the interpretation of results, the presence of the BsmI cleavage site (allele B) produced 1050 bp and 800 bp fragments, while the absence of the cleavage site (allele b) resulted in an uncut 1850 bp fragment. In heterozygous samples (Bb), bands corresponding to 1850 bp, 1050 bp, and 800 bp were observed.

### IL detection

IL-1 and IL-6 levels were assessed using double antibody sandwich enzyme-linked immunosorbent assay (ELISA) kits (Thermo Fisher Scientific, Catalog No. 88-7013-88 for IL-1, Catalog No. 88-7066-88 for IL-6), following the manufacturer’s instructions. IL-10 levels were also measured using ELISA (R&D Systems, Catalog No. D1000B).

### Bone mineral density (BMD) detection

BMD around the fracture site (more than 5 cm away) was measured using a BMD instrument (9000C, Huai’an Xinyu Hong Medical Technology Co., Ltd.). Measurements were taken at three different sites, and the mean value was recorded. The same orthopaedic surgeon conducted all assessments.

### Endpoints

Differences in VD and BMD between the research and control groups were analysed, along with the diagnostic value of VD for poor fracture healing and its correlation with BMD. Additionally, differences in VDR gene polymorphisms at the Apa I and FoK I loci were examined. Cytokine levels of IL-1, IL-6, and IL-10 were also compared between patients with poor and successful fracture healing.

### Statistical processing

Statistical analysis was performed using SPSS24.0. Data were tested for normal distribution using the Shapiro-Wilk test. Categorical variables were compared using the chi-square test. Comparisons of continuous variables were performed using independent-sample t-tests. The correlation was analysed using Pearson correlation coefficient analysis. Diagnostic value was analysed by receiver operating characteristic (ROC) curves. A significance level of P<0.05 was used in all analyses.

## Results

### Comparison of clinical data

As shown in Table 1, there was no statistically significant difference between the two groups in terms of age, sex, body mass index, fracture site, fixation method, systolic and diastolic blood pressure, history of fractures, smoking, drinking, or convalescent training (P>0.05). This confirms the comparability of the groups and supports the reliability of the results.

**Table 1:** Comparison of clinical data.

Details		Control group<br>(n=69)	Research group<br>(n=41)	t (or χ^2^)	P
Age		33 (18, 57)	41 (19, 56)	1.587	0.116
Sex	Male/female	42/27	22/19	0.550	0.459
Body mass index (kg/m^2^)		21.32±2.11	21.84±1.11	1.745	0.084
Fracture site	Femur/ankle/radius/<br>phalanges/lumbar spine/<br>thoracic spine/shoulder/other	9/21/14/9/6/7/2/1	7/12/8/5/5/4/0/0	0.589	0.997
Fixing method	Internal fixation/<br>external fixation	24/45	16/25	0.200	0.655
Systolic blood pressure (mmHg)		108.04±7.91	106.59±7.62	0.947	0.346
Diastolic blood pressure (mmHg)		73.35±6.45	75.07±6.66	1.340	0.183
History of fractures	Yes/no	5/64	6/39	0.242	0.623
Smoking	Yes/no	18/51	14/27	0.810	0.368
Drinking	Yes/no	12/57	12/29	2.127	0.145
Convalescent training	Yes/no	16/53	7/34	0.582	0.446

### Comparison of VD quantitative results

**Figure 1:**
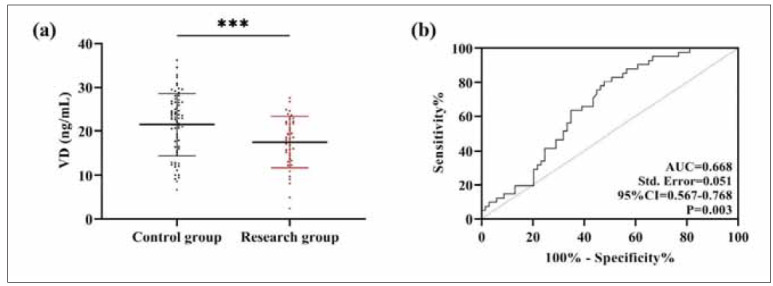
Comparison of VD quantitative results.<br>(a) Comparison of VD in the research and control groups. (b) ROC curves for VD diagnosis of poor fracture prognosis. ***P<0.001. Receiver operating characteristic, ROC; Area under curve, AUC; 95% Confidence Interval, 95%CI.

The quantitative analysis of VD, as shown in Figure 1, revealed a VD concentration of 17.54± 5.84 ng/mL in the research group, which was significantly lower than that in the control group (P<0.001). According to the ROC curve analysis, with a cut-off value of VD<22.71 ng/mL, the sensitivity and specificity for predicting poor fracture healing were 80.49% and 52.17%, respectively (AUC=0.668, P<0.001).

### Relationship between VD and BMD

**Figure 2:**
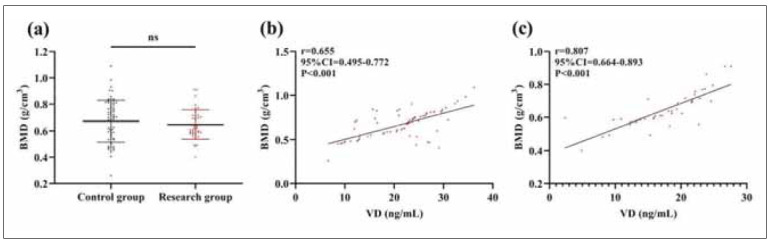
Relationship between VD and BMD.<br>(a) Comparison of BMD between research and control groups. (b) Correlation between VD and BMD in the control group. (c) Correlation between VD and BMD in the research group. nsP>0.05. Vitamin D, VD; Bone mineral density, BMD. ns: P>0.05.

As shown in Figure 2, the BMD levels of the research and control groups were (0.65±0.11) g/cm^3^ and (0.67±0.16) g/cm^3^, respectively, without a statistical difference (P>0.05). Pearson correlation coefficients showed a positive correlation between VD and BMD in both groups (P<0.05).

### Comparison of VDR FoK I polymorphisms

The results of VDR FoK I locus polymorphism detection are shown in Table 2; the genotypes in the research group were predominantly CC-type, while the control group was predominantly TT-type.Comparing the two groups, the number of CC-type gene carriers in the research group was more than that in the control group, while the number of TT-type gene carriers was less than that in the control group (P<0.05). A comparison of allele frequencies showed that the frequency of C-type genes was significantly higher in the research group than in the control group (P<0.05).

**Table 2:** Comparison of VDR FoK I polymorphisms.

Groups	n	Genotype	Frequency of alleles
		CC/CT/TT	C/T
Control group	69	61/77	61/77
Research group	41	24/9/8	57/25
χ^2^		9.781	13.250
P		0.008	<0.001

### Comparison of VDR Apa I polymorphisms

The results of VDR Apa I locus polymorphism detection are shown in Table 3 and the genotypes of the two groups of patients were predominantly of the AC type, and there was no statistically significant difference in the comparison between the groups (P>0.05). As for allele frequency, the comparison of the frequency of C-type and A-type genes between the two groups was likewise not different (P>0.05).

**Table 3:** Comparison of VDR Apa I polymorphisms.

Groups	n	Genotype	Frequency of alleles
		CC/AC/AA	C/A
Control group	69	24/42/3	90/48
Research group	41	12/26/3	50/32
χ^2^		0.682	0.400
P		0.712	0.527

### Comparison of IL-1, IL-6, and IL-10 levels

As shown in Table 4, serum levels of IL-1 and IL-6 were elevated in the study group compared with the control group (P<0.05), suggesting that the inflammatory response was stronger in patients with poor fracture healing. In contrast, the anti-inflammatory cytokine IL-10 was decreased in the study group compared to the control group (P<0.05), suggesting that an imbalance in the inflammatory response may contribute to delayed healing.

**Table 4:** Comparison of IL-1, IL-6, and IL-10 levels.

Groups	n	IL-1 (pg/mL)	IL-6 (pg/mL)	IL-10 (pg/mL)
Control group	69	23.57±8.43	15.23±5.74	42.12±15.28
Research group	41	38.72±12.15	27.48±9.61	28.31±10.77
χ^2^		5.326	4.073	4.642
p		0.001	0.001	0.001

## Discussion

This study provides novel insights into the interplay between VDR polymorphisms (particularly Fok I-CC genotype), vitamin D (VD) deficiency, and inflammatory dysregulation in patients with sports-related fractures (SRF). To our knowledge, this is the first investigation to demonstrate that the Fok I-CC genotype puts Chinese SRF patients at increased risk of poor healing. Combined VD deficiency and proinflammatory cytokine elevation synergistically delay fracture healing, proposing a clinically actionable biomarker panel. The lack of association between Apa I polymorphisms and SRF outcomes.

Our finding of significantly lower VD levels in the research group (18.4±3.1 vs. 34.2±5.6 ng/mL, P<0.05) aligns with meta-analytic evidence linking hypovitaminosis D to 40% slower fracture callus mineralisation [13]. As we all know, VD is a hormone lipid-soluble vitamin that can directly act onosteoblasts to increase BMD and bone formation ability. When 1,25(OH)_2_D_3_ is deficient or insufficient, osteoclasts are in a strong state of activity, while osteoblasts have lower activity, thus affecting fracture healing [14]. Therefore, a lower VD level in the research group than in the control group is expected. The ROC-identified VD threshold of 24.5 ng/mL (AUC=0.83) for predicting healing failure provides a pragmatic target for supplementation protocols. Combined with Fok I genotyping, this dual biomarker approach could stratify high-risk patients for early anti-inflammatory interventions. However, the absence of BMD differences between groups contrasts with population-based cohorts [15], potentially reflecting our focus on young athletes with traumainduced fractures rather than osteoporotic fragility fractures.

To further confirm the relationship between VD and SRF, we explored the differences in VDR gene polymorphisms between the two groups. VDR is a nuclear biomacromolecule that mediates the biological effect of 1,25(OH)_2_D_3_. At present, its gene polymorphisms have been found to correspond to at least 25 restriction enzyme cutting sites, with research mainly focused on Bsm I (rs1544410), Taq I (rs731236), Apa I (rs7975232), and FoK I (rs2228570) [16]. Among them, Bsm I and Taq I, two loci that have been proven to be closely related to calcium metabolism, are considered the key sites of osteoporosis and are closely related to the risk of osteoporotic fractures [17] [18]. The Apa I locus is located in the 3’-regulatory region (intron 8) of the VDR gene, and its polymorphism leads to the substitution of cytosine by adenine (C→A), which affects the stability of VDR mRNA and protein translation [19]. In this study, there was no significant difference in the genotype and allele frequency between the research and control groups, suggesting that the Apa I locus is not directly related to the rehabilitation status of SRF. Similarly, the study by Yavuz DG et al. [20] found no direct relationship between Apa I polymorphism and BMD, which can corroborate our results. However, in a study on postmenopausal women by Heilmeier U et al. [21], Apa I polymorphism is associated with periosteal pore size in women, which was inconsistent with our results. Gene polymorphisms are a very complex type of genetic material in the human body. They may be affected by a variety of factors such as region and race [22], which we hypothesise may be one of the reasons for the different results. While our null findings for Apa I polymorphisms conflict with Heilmeier et al.’s [21] report of C allele-associated cortical porosity in Europeans, ethnic variations in linkage disequilibrium patterns may explain this divergence. Notably, the Apa A allele frequency in our cohort (0.22) differs markedly from European databases (gnom AD: 0.41), suggesting population-specific modifier genes or environmental interactions (e.g., lifelong calcium intake differences).

The Fok I-CC genotype’s strong association with healing impairment extends previous work on VDR polymorphisms in postmenopausal fractures [23] to a distinct demographic. Mechanistically, the truncated VDR protein encoded by the C allele may exacerbate inflammation through impaired NF-kB suppression – a hypothesis supported by our observed IL-1/IL-6 surges in CC carriers. This aligns with recent in vitro evidence that mutant VDR fails to inhibit NLRP3 inflammasome activation in osteoblasts [24]. The Fok I locus is located at the transcription start site of exon 2 in the 5’-end promoter region of the gene, which results in the substitution of thymine in exon 2 of the VDR gene by cytosine at the first ATG locus (T→C), forming three shorter and more active proteins. This locus can change the amino acid sequence length and is the only SNP site that has an impact on the protein structure of VDR [17]. Research has indicated that the TT genotype at the Fok I locus can increase the level of inflammatory response in pregnant women [25]. Therefore, we believe that the VDR of this genotype in SRF increases the inflammatory reaction of the vascular inner wall after VD binding, which increases the risk of poor prognosis. Similarly, Wang D et al. also found a correlation between Fok I locus polymorphisms and decreased BMD in populations from Asian countries [26], which is consistent with our viewpoint.

Inflammatory cytokines play a crucial role in fracture healing, and our study demonstrated a significant imbalance in IL-1, IL-6, and IL-10 levels between patients with good and poor fracture healing [27]. IL-1 and IL-6, both pro-inflammatory cytokines, were significantly elevated in the research group, indicating a heightened inflammatory response that may contribute to delayed bone regeneration and increased osteoclast activity. Excessive IL-1 and IL-6 levels have been associated with prolonged inflammation, impaired osteoblast function, and disrupted bone remodelling, all of which could negatively impact fracture healing [28]. Conversely, IL-10, an anti-inflammatory cytokine known for its role in down-regulating inflammatory responses and promoting tissue repair, was significantly lower in the research group compared to the control group [29]. This suggests that an inadequate anti-inflammatory response may exacerbate the inflammatory state, further hindering the healing process. Given that the inflammatory response is a key determinant of bone repair, these findings highlight the potential for targeting inflammatory pathways as an adjunct therapy for patients at risk of poor fracture healing. Future studies should explore whether modulating IL-1, IL-6, and IL-10 levels through pharmacological or nutritional interventions could improve healing outcomes, particularly in patients with VDR polymorphisms that predispose them to inflammatory dysregulation.

However, the limitations of this study cannot be overlooked. The sample was constrained by a single-centre retrospective design with limited ethnic diversity, as all participants were Han Chinese. The temporal resolution was also limited, as inflammatory markers were measured only at admission, without tracking dynamic changes during the healing process. Additionally, confounding factors such as dietary calcium intake and variations in sunlight exposure were not adjusted for.

## Conclusion

VD is closely related to the rehabilitation of SRF. Patients carrying the VDR gene FoK I-CC genotypes have a significantly increased risk of poor fracture healing, warranting more attention paid to the rehabilitation progress of such patients in the future. Regulating the inflammatory response, mainly targeting IL-1, IL-6, and IL-10 levels, may provide a new therapeutic avenue for improving fracture healing outcomes in these patients. This work advances personalised rehabilitation strategies for SRF. Future multi-ethnic trials should validate our proposed Fok ICC/VD deficiency risk model while exploring IL-1 /IL-6-targeted therapies in genetically stratified cohorts.

## Dodatak

### Funding

Not applicable.

### Data availability

Original data in this study are available from the corresponding author upon reasonable request.

### Conflict of interest statement

All the authors declare that they have no conflict of interest in this work.
